# DAMP-driven trained immunity: metabolic and epigenetic reprogramming in critical illness and chronic inflammation

**DOI:** 10.3389/fimmu.2025.1669054

**Published:** 2025-11-24

**Authors:** Han G. Kim, Jaimar C. Rincon, Philip A. Efron, Robert Maile

**Affiliations:** Sepsis and Critical Illness Research Center, Department of Surgery, College of Medicine, University of Florida College of Medicine, Gainesville, FL, United States

**Keywords:** innate training, DAMPs, trauma, epigenetics, innate immunity

## Abstract

Innate immune memory, traditionally underappreciated in contrast to adaptive immunity, is now recognized as a critical component of host defense, particularly in the context of sepsis and sterile inflammatory injury. Recent advances have identified a central role for metabolic and epigenetic reprogramming in driving trained immunity (TRIM), where monocytes, macrophages, and other innate cells develop enhanced or tolerized responses to secondary stimuli. This review synthesizes current knowledge of how damage-associated molecular patterns (DAMPs), including oxidized LDL, HMGB1, heme, urate crystals, and mitochondrial DNA, serve as potent inducers of immunometabolic rewiring, often through the mTOR/HIF-1α axis or alternative pathways such as SYK signaling. We highlight distinct epigenetic mechanisms, such as enhancer priming via H3K4me1/H3K27ac, and metabolic shifts like the Warburg effect, succinate accumulation, and fatty acid synthesis, that define the trained or tolerized states. Particular attention is given to the relevance of these mechanisms in the pathophysiology of sepsis, burns, trauma, and other critical illnesses where persistent DAMP exposure may sustain maladaptive inflammation or immunosuppression. We review data linking central (stem cell-level) and peripheral reprogramming to long-term immune dysfunction in various inflammatory disease models, and explore how DAMPs intersect with PAMPs to shape the immune trajectory. Finally, we identify pressing gaps in the field, including the need for standardized TRIM models, validated biomarkers of innate memory, and mechanistic clarity on mitochondrial DAMPs in immune tolerance. These insights provide a foundation for future therapeutic strategies aimed at modulating trained immunity to improve outcomes in critically ill patients.

## 1 Introduction

**Figure 1:**
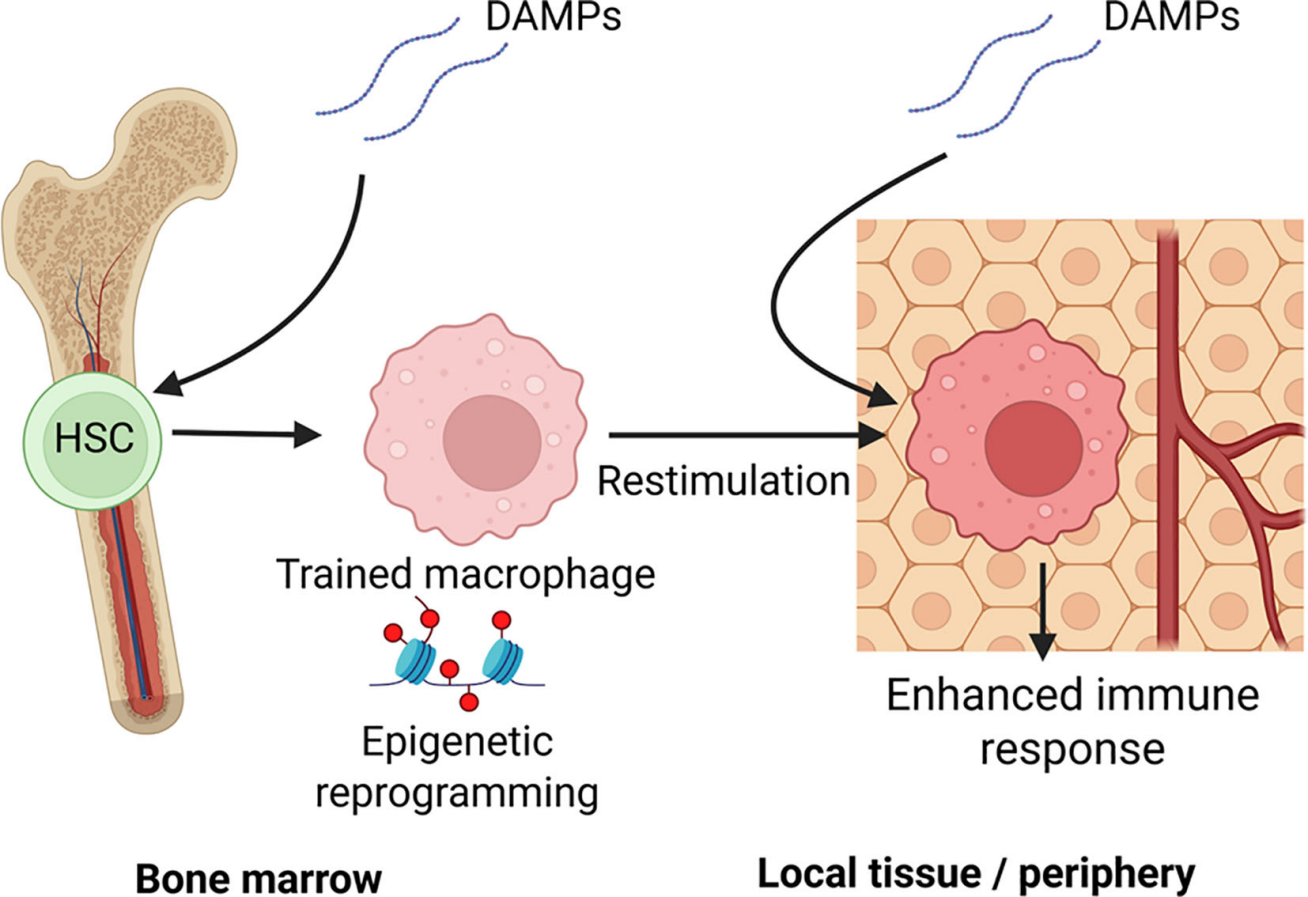
Central and peripheral mechanisms of DAMP-induced innate immune memory: This schematic illustrates how damage-associated molecular patterns (DAMPs), such as those released during trauma, burns, or ischemic injury, can induce trained immunity through both central and peripheral pathways: Central Training (left): DAMPs reaching the bone marrow microenvironment activate pattern recognition receptors (e.g., via IL-1β or systemic oxidized lipids), leading to epigenetic reprogramming of hematopoietic stem and progenitor cells (HSPCs). These changes imprint a trained phenotype in progeny monocytes and macrophages, skewing hematopoiesis toward a myeloid-biased, pro-inflammatory lineage. Peripheral Training (right): In local tissues, resident or recruited monocytes and macrophages encounter DAMPs (e.g., heme, HMGB1, uric acid) and undergo metabolic and epigenetic reprogramming. Upon secondary stimulation (e.g., by infection or reinjury), these cells exhibit altered immune responses; in the figure, trained immunity is enhanced cytokine production, ROS generation, and antimicrobial activity. Together, these establish a durable innate memory state that influences systemic immunity long after the initial DAMP exposure has resolved. Created with BioRender.com.

Traditionally, immune memory is associated with clonal expansion and antigen specificity that is marked by adaptive immune responses. Meanwhile, the innate immune system has been understood as a nonspecific first line of defense that does not involve immunological memory. Despite a lack of antigen specificity, innate cells exhibit germline-encoded pattern recognition receptors (PRRs) that are able to recognize both Pathogen-Associated Molecular Patterns (PAMPs) as well as Damage-Associated Molecular Patterns (DAMPs). Recent research has demonstrated that these signaling pathways are linked to metabolic/epigenetic rewiring that results in not only short-term peripheral changes in innate immunological function, but also long-term central myeloid-based changes (1) (**Figure 1**).

**Figure 2:**
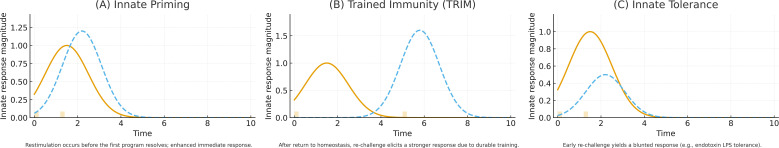
Three forms of innate immunological memory are elicited by common PRR activation and epigenetic/metabolic rewiring but differ in kinetics and functional outcome. **(A)** Innate priming is transient; re-stimulation before completion of the first transcriptional program yields an enhanced immediate response (orange line, primary response; blue dotted line, re-challenge response). **(B)** TRIM persists beyond return to metabolic homeostasis, enabling an augmented response to delayed re-challenge. **(C)** Innate tolerance occurs on a similar timescale to priming, but early re-stimulation produces a blunted response; endotoxin (LPS) tolerization is a canonical example.

Three forms of innate immunological memory have now been described: priming, training, and tolerance characterized by distinct function and duration despite common activation using PRRs and epigenetic/metabolic rewiring (**Figure 2**). Innate priming describes a transient state where restimulation occurs prior to the end of the first stimulation, resulting in an enhanced response. This contrasts with trained immunity (TRIM) where even after the cell returns to metabolic homeostasis, it is able to present an enhanced immune response. Finally, there is innate tolerance which is similar to priming in time-scale, but restimulation results in a decreased ability to mount an immune response. Endotoxin LPS tolerization is a common example for this form of innate memory.

Despite differences in presentation, all three types of memory share common underlying mechanisms as evidenced by the ability of one memory type to influence the other. For example, *C. albicans* cell wall component β-glucan has been shown to reverse epigenetic reprogramming by LPS-tolerization resulting in cells being able to produce robust pro-inflammatory cytokines following LPS re-exposure (2). Studies like these illustrate a need to understand innate memory mechanisms while better defining pathways leading to specific memory responses.

One of the earliest works demonstrating the link between innate memory and epigenetics came from a 2007 study on LPS tolerance. Toll-like Receptor (TLR)-4 mediated LPS signaling of peripheral macrophages induced two gene types with differentiated epigenetic phenotypes; genes that are silenced (tolerizeable) and genes that can be primed (non-tolerizeable) (3). The concept of long-lasting TRIM first began from observations of viral vaccines such as Bacile Calmette-Guérin (BCG) resulting in reduced patient mortality in non-targeted pathogens for months after initial vaccination (4). Further research into BCG and microbial ligands like β-glucan reveal functional reprogramming resulting in nonspecific enhancement of the innate immune system’s ability to respond to subsequent stimuli (5–7). However, this innate functional reprogramming is not exclusive to PAMPs but have also been noted in endogenous DAMPs. For example, oxidized low-density lipoproteins (oxLDL), heme, and vimentin have all been connected to TRIM reprogramming in both autoimmune and sepsis/trauma related disease models (8, 9). Nevertheless, despite evidence of endogenous molecules as inducers of innate training, the difference in mechanism between PAMPs and DAMPs and their coordinated function is still poorly understood.

A fundamental concern in the field is that trained immunity and tolerance lack unified experimental criteria. Different groups use different primary stimuli (from LPS to β-glucan to necrotic cell extracts) and different secondary challenge assays to define “memory.” As a result, what is labeled as trained immunity in one study might not fully match another’s conditions. There are also contradictory findings; for example, some studies report that tolerized macrophages have diminished pathogen killing ability, while others show tolerized cells retaining or even enhancing certain antimicrobial functions. These discrepancies point to unknown modulators (such as cell type, species, or timing differences) that are not accounted for. The lack of standardized models is a problem: without common benchmarks, it is fundamentally difficult to compare results and build a coherent theory. This issue underlies many debates and slows progress in the field.

To understand the role of DAMPs in innate memory, we will outline the key mechanistic underpinnings of innate memory (epigenetics and metabolic changes), and then explore various DAMP-related innate immune training literature to define the current landscape. We will then discuss future directions of study of DAMP-related innate memory.

## 2 The role of DAMPs in inflammation

DAMPs were first recognized by Polly Matzinger as alarm signals of cellular distress that work alongside pathogen-associated pathways within the immune system (10). These ‘alarmin’ signals can be triggered by various stressors such as radiation, oxidative stress, and chemical agents (11). Both DAMPs and PAMPs act on PRRs which are germ-line proteins that recognize common structures found on bacteria to initiate inflammatory responses (12). There are four main subcategories of PRRs which TLR, Nucleotide-binding oligomerization domain (NOD)- Leucine rich repeat (LRR)-containing receptors (NLRs), Retinoic acid-inducible gene I (RIG-I)-like receptors as well as C-type lectin receptors (CLRs) (13).

In steady-state conditions, these endogenous molecules are thought to not induce inflammatory pathways. However, under infection, stress, or injury, these molecules can be converted into DAMPs *via* three mechanisms as recently described: displacement from a tolerant position, change of chemical properties, or change in the molecule concentration (14). Generally, DAMPs serve a beneficial role by upregulating immune defense, maintenance of homeostasis, and tissue repair. However, they also facilitate continual upregulation of inflammatory signals that, when pushed to extremes, can result in immune dysfunction. For example, DAMPs like HMGB1 and cell free DNA (cfDNA) from neutrophil NETosis have been correlated with sepsis severity (15–17) Furthermore, DAMPs can play a role in initiating autoimmune conditions with multiple sclerosis studies highlighting the role that high-mobility group box 1 (HMGB1) and heat shock protein 70 (HSP70) play in autoimmune encephalomyelitis (18–20). Moreover, extensive research has been conducted on the DAMP oxLDL for its ability to induce NLRP3-mediated TRIM (21).

However, it is important to note that despite many commonalities in DAMP and PAMP-mediated pathways, there are notable differences in how they interact with the immune system. Although the DAMP HMGB1 has been linked to LPS-mediated TLR4 desensitization (22), studies note DAMPs alone may not be enough to completely inhibit TLR signaling (23). Therefore, DAMPs likely play a synergistic role with PAMPs. For example, the DAMP oxPAPC, the bioactive component of oxLDL, when coactivated with LPS elicits hyperinflammatory metabolic changes compared to isolated treatments (24). Interestingly, although studies involving oxLDL reveal similar training mechanisms to PAMPs like β-glucan, other DAMPs such as heme present divergent pathways resulting in TRIM (8, 9). Furthermore, recent research has proposed alternative methods of endogenous damage signaling. For example, research into extracellular vesicles (EVs) have shown that EVs serve as mediators of inflammatory responses in various traumatic injuries and sepsis (reviewed in (25)). In addition to miRNAs, proteins, mRNA, and lipids, burn EVs possess DAMPs such as HMGB1 (26). Moreover, transfer of EVs isolated from burn patients or mice can induce immunological dysfunction (27–29). These studies illustrate that further research needs to be done to outline key mechanisms behind DAMP’s ability to induce TRIM in disease-relevant models.

## 3 Central *versus* peripheral differences in training

Innate memory exists at the level of the local tissue through “peripheral training” while “central training” occurs at the level of Hematopoietic Stem and Progenitor cells (HSPC) such as myeloid progenitor cells in the bone marrow (**Figure 1**). In BCG-vaccination studies, induction of BCG into bone marrow can induce epigenetically modified macrophages with improved clearance of pathogens (30). However, central reprogramming are not limited to PAMP activity. Duchenne muscular dystrophy (DMD) is a fatal genetic disease linked to trained immunity. In a study using adoptive transfer of bone marrow-derived macrophages from DMD mice to control mice, hyperreactive macrophages could last up to 11 weeks post-transplant (31). DAMPs such as heme can activate hematopoietic stem cells in the bone marrow resulting in both increased percentage and frequency of myeloid lineage differentiation correlating with TRIM-related epigenetic changes and *in-vivo* resistance to bacterial sepsis (9). Finally, a recent study linked a transient occlusion of the middle cerebral artery to upregulated TRIM responses that persisted in monocytes and macrophages for up to 3 months post-injury (32). This was linked to epigenetic remodeling of bone marrow derived cells, establishing a link between neurological injury to cardiac dysfunction and highlighting a clinical application of DAMP induced training on central immune processes.

## 4 Epigenetic and metabolic rewiring in the context of innate memory

Divergent innate immune memory states are induced by PAMPs and DAMPs through metabolic and epigenetic reprogramming. In a seminal study, mice lacking T and B cells were protected against reinfection with Candida albicans through monocyte-dependent mechanisms, introducing the term “trained immunity” and linking it to promoter H3K4me3 gains at inflammatory genes (5). Integrative epigenome-transcriptome profiling later showed that monocyte-to-macrophage differentiation and trained immunity involve coordinated promoter and enhancer remodeling, with β-glucan training co-inducing H3K4me3 at promoters and H3K4me1/H3K27ac at distal enhancers (33) Complementing this, β-glucan could partially reverse LPS-induced tolerance in humans and restore cytokine production, underscoring distinct epigenetic routes to training versus tolerance (2).

**Figure 3:**
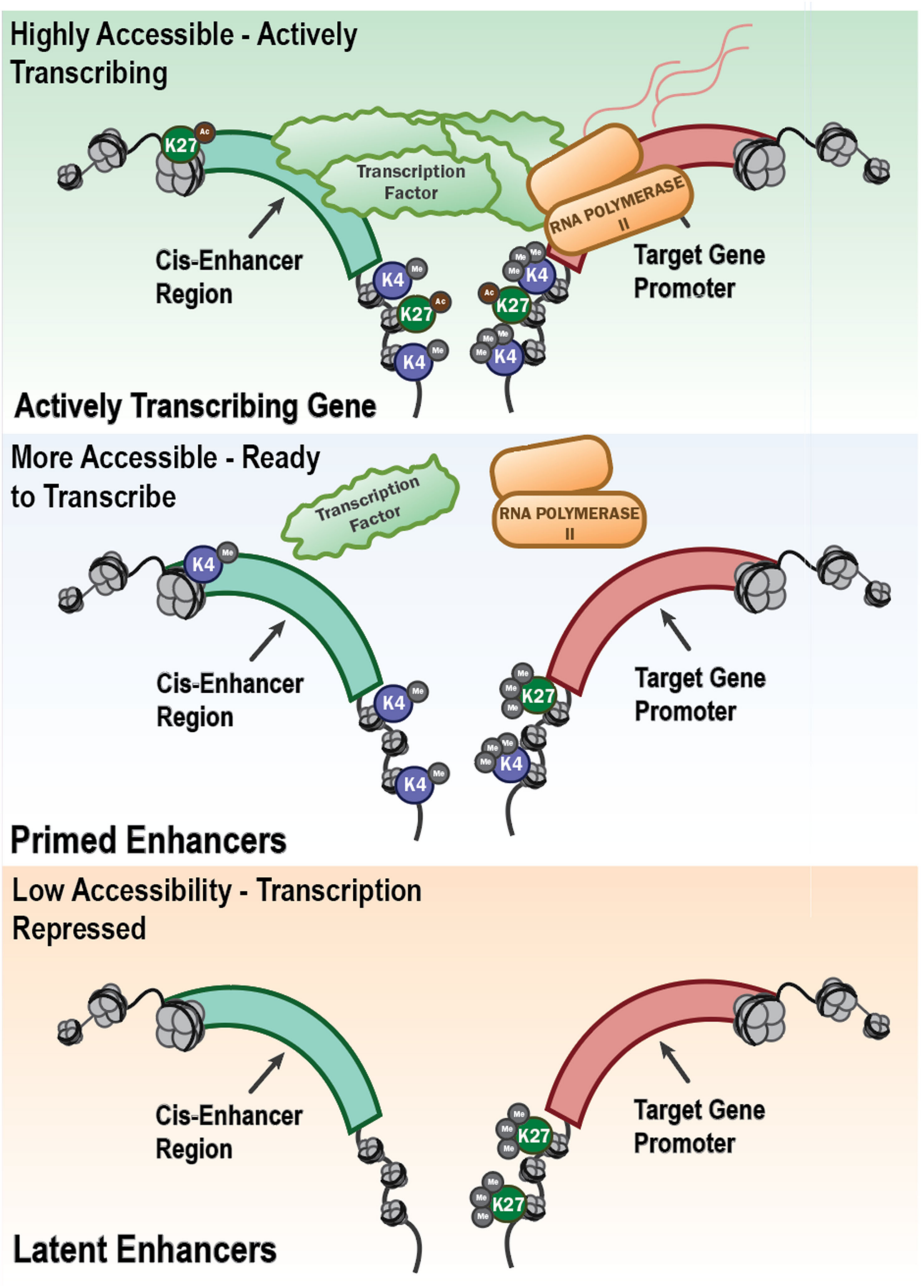
Epigenetic regulation of gene transcription through enhancer accessibility and histone modifications. This schematic illustrates the progressive chromatin states that define the transcriptional potential of innate immune genes. Open chromatin with active histone modifications (e.g., H3K4me3 and H3K27ac) at both the cis-enhancer region and gene promoter allows recruitment of transcription factors and RNA polymerase II, resulting in active transcription. This is shown as “Highly Accessible – Actively Transcribing” (top panel) in the schematic. Other regions, “Primed Enhancers” (middle panel) are regions that are more accessible and marked by H3K4me1 at enhancers and H3K4me3 at promoters, with limited or absent H3K27 acetylation. They are poised for rapid activation but not actively transcribing, representing a key feature of trained innate immunity. In contrast, “Latent Enhancers” (bottom panel) have closed chromatin marked by H3K27me3 or a lack of activating histone marks which are associated with low accessibility and transcriptional repression. These regions may become activated under specific stimuli or remain epigenetically silenced, characteristic of immune tolerance. Together, these chromatin states underlie the long-term transcriptional reprogramming that defines trained immunity or tolerance in innate immune cells.

These observations fit a promoter-enhancer framework in which H3K4me3 marks active promoters, while H3K4me1 and H3K27ac demarcate primed and active enhancers, respectively (34)(**Figure 3**) The “latent enhancer” model posits that previously unmarked regions acquire transcription factor (TF) binding and enhancer marks upon stimulation and then respond faster upon restimulation (35) Functionally, enhancer acetylation can amplify transcription by modulating burst kinetics, providing a causal link between H3K27ac and increased output (36, 37). Causality is further supported by perturbations of chromatin enzymes. Inhibiting the H3K9 methyltransferase G9a (EHMT2) decreased H3K9me2 at promoters and augmented trained responses to diverse stimuli in human monocytes and in patients receiving BCG (38). Together, these studies support a concise model: β-glucan-induced trained immunity is encoded by coordinated promoter and enhancer rewiring. This is centered on H3K4me3 at promoters and H3K4me1/H3K27ac at enhancers, whose functional consequence is enhanced transcriptional bursting and cytokine output upon rechallenge (reviewed in (39). Non-coding RNAs (ncRNAs) act as upstream “scaffolds” and downstream “switches” that couple metabolism to chromatin in TRIM. A landmark human macrophage study identified UMLILO, a proximal lncRNA at the chemokine locus, which recruits chromatin-modifying machinery to install H3K4me3/H3K27ac and prime CXCL promoters for rapid re-activation. This directly demonstrated an lncRNA requirement for trained responses (1). At the microRNA (miRNA) scale, in β-glucan-trained monocytes, miR-9-5p targets IDH3A, lowers α-KG and favors fumarate accumulation, thereby reinforcing HIF-1α-linked glycolysis and histone methylation that encode training. Genetic loss of miR-9-5p blunted TRIM cytokines *in vitro* and impairs trained responses *in vivo (*40). By contrast, miR-146a dampens TLR signaling and is necessary for endotoxin tolerance in human monocytic models (41). These data illustrate how distinct ncRNAs drive trained *versus* tolerized trajectories.

**Figure 4:**
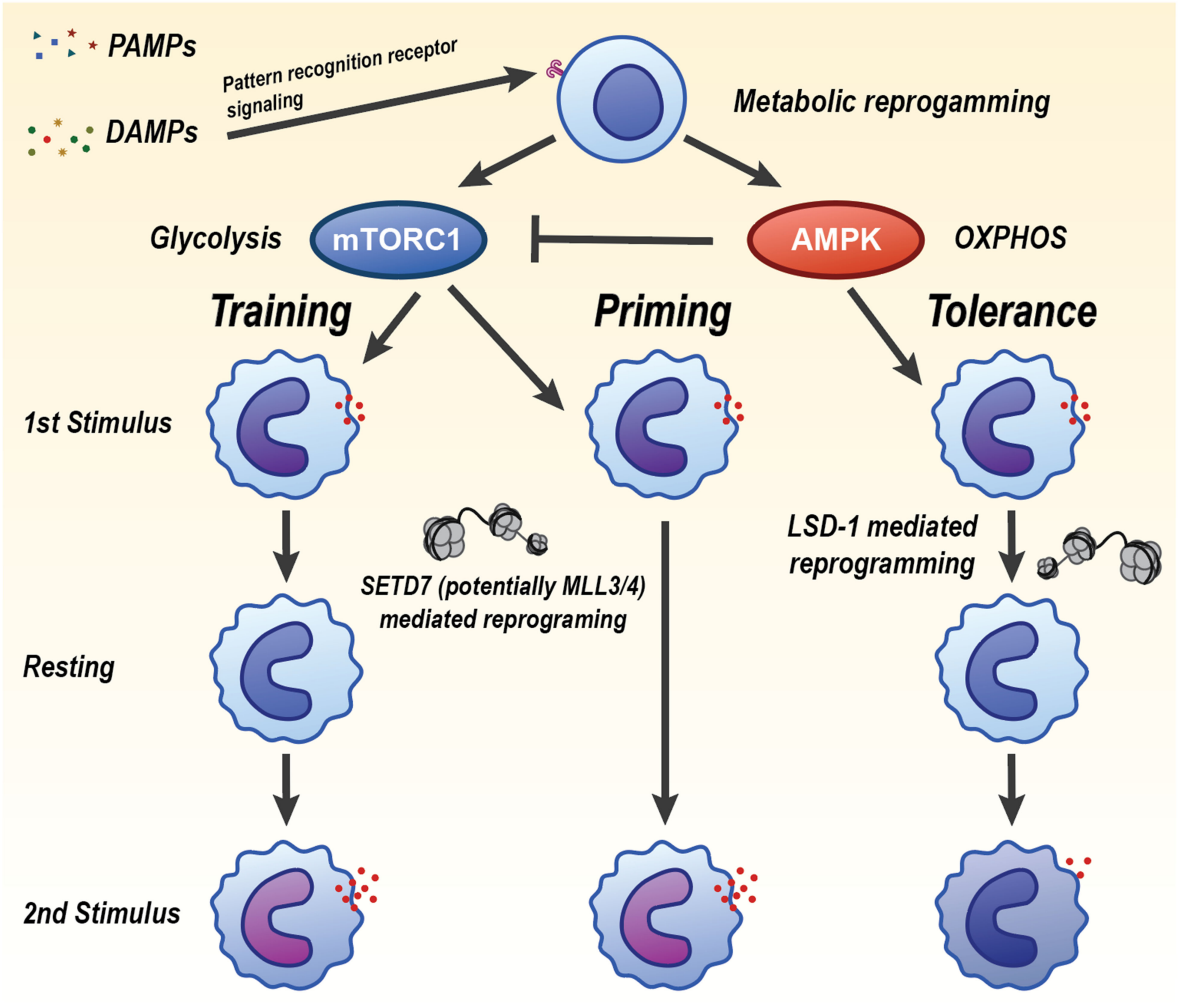
Divergent innate immune memory states induced by PAMPs and DAMPs through metabolic and epigenetic reprogramming. Pathogen-associated molecular patterns (PAMPs) and damage-associated molecular patterns (DAMPs) signal through pattern recognition receptors to initiate metabolic reprogramming in innate immune cells. The balance of mTORC1 and AMPK activity may determine whether the cell undergoes trained immunity, priming, or tolerance. Trained immunity is driven by mTORC1 activation and increased glycolysis, leading to heightened inflammatory responses upon secondary challenge. This is maintained by SETD7- and possibly MLL3/4-mediated epigenetic reprogramming. Tolerance is characterized by AMPK-driven oxidative phosphorylation (OXPHOS), reduced inflammatory cytokine production, and is reinforced by LSD1-mediated chromatin remodeling. Priming represents an intermediate or poised state that can resolve into training or tolerance depending on context and secondary cues. Each state is associated with a distinct transcriptional and functional response upon restimulation, regulated by metabolic pathways and histone-modifying enzymes that mediate long-term gene expression changes.

Upon primary stimulation, monocytes and macrophages also undergo a rapid shift toward aerobic glycolysis with increased glucose uptake and lactate production, which supports ATP generation and biosynthesis required for heightened cytokine output during rechallenge (**Figure 4**). A seminal mechanistic study established that β-glucan training is driven by the Phosphoinositide 3-kinase (PI3K)/protein kinase B (Akt)/mechanistic target of rapamycin (mTOR) and Hypoxia-Inducible Factor 1α (HIF-1α) axis that installs a glycolytic program; pharmacologic or genetic inhibition of AKT, mTOR, or HIF-1α abrogated both the metabolic shift and trained cytokine responses *in vitro* and *in vivo* (42). Consistent human and vaccination data show that BCG training elevates glycolytic flux and glucose uptake, with mTOR as an upstream controller (43). Beyond glycolysis, rewiring of the tricarboxylic-acid network provides signaling metabolites that couple metabolism to chromatin. In β-glucan-trained monocytes, glutaminolysis-driven accumulation of fumarate integrates immunometabolic and epigenetic programs by inhibiting KDM5 histone demethylases. Exogenous fumarate alone can imprint a trained phenotype, whereas blockade of glutaminolysis or cholesterol synthesis impairs training in mice (44). In parallel, the mevalonate branch of cholesterol biosynthesis acts as a pro-training signal: mevalonate activates insulin-like growth factor 1 receptor (IGF1R)-mTOR and promotes histone modifications at inflammatory loci. Statins prevent induction of trained immunity, while mevalonate kinase deficiency is associated with a constitutively “trained” state (45). Diet and sterile DAMPs can similarly program innate memory; notably, short-term Western diet triggers NLRP3-dependent trained immunity and myelopoietic skewing *in vivo* (21). Tolerance represents a counter-programmed metabolic state characterized by suppression of glycolysis and reliance on oxidative phosphorylation and fatty-acid oxidation, often enforced by anti-inflammatory mediators and stress sensors. Mechanistically, the itaconate pathway limits inflammatory output and contributes to tolerization by directly inhibiting Ten-eleven translocation (TET) DNA dioxygenases, thereby reshaping the epigenome and dampening NF-κB/STAT programs (46). Under glucose restriction or settings favoring catabolism, monocytes compensate with AMPK-driven fatty-acid oxidation and mitochondrial respiration, which sustains core functions while reducing respiratory burst (47) Together, these studies define trained immunity as a coordinated metabolic program centered on mTOR-dependent glycolysis, reinforced by Tricarboxylic Acid Cycle (TCA)-derived signaling metabolites and the mevalonate pathway, that licenses durable functional reprogramming of innate cells. The tolerant trajectory instead engages itaconate-TET2 signaling and AMPK-supported oxidative metabolism to constrain inflammatory outputs.

## 5 Specific DAMPs as modulators of innate memory

DAMPs-mediated innate memory compared to PAMPs, is poorly understood with limited research in its role on both short-term priming and long-term immunometabolic reprogramming. Many studies have since explored DAMPs’ role in TRIM while outlining metabolic and epigenetic reprogramming of both priming and TRIM (48) (**Table 1**). Recent studies into PAMPs also demonstrate a synergy in PAMP-driven TRIM responses enhanced by the release of DAMPs (49).

**Table 1:** Key pattern recognition receptors, metabolic programs, and epigenetic mechanisms involved in DAMP-induced trained immunity.

Training-relevant DAMP	TLR signaling	Metabolic pathway	Epigenetic pathway	PMID References
oxLDL	TLR4/TLR6 (via CD36), TLR2	Akt/mTOR/HIF-1α, glycolysis, mevalonate	H3K4me3/H3K27ac; MTA/EGCG inhibitable	(21, 33, 127)
HMGB1	TLR2, TLR4, RAGE	Dose-dependent; mTOR activation (acute), SIRT1/AMPK (chronic)	H3K27ac; EV-mediated, unclear	(128–131)
Vimentin	Dectin-1	mTOR-dependent via Dectin-1	H3K27ac via mTOR; rapamycin-sensitive	(132, 133)
Hyperglycemia	N/A (indirect via glucose sensing)	Increased glycolysis, HIF-1α activation	MLL3/4; H3K4me3 enrichment	(45, 75, 134)
Heme	TLR4, NLRP3	Syk/ROS pathway, mTOR-independent	H3K27ac; LSD1 sensitive, mTOR-independent	(77, 135, 136)
Urate/Uric Acid	TLR2, TLR4 (via MSU), NLRP3	mTOR activation, NLRP3, glycolysis	H3K4me3/H3K27ac; HDAC-sensitive	(80, 137)
mtDNA	TLR9 (CpG motifs)	STING/autophagy → mTOR inhibition	CpG-mediated training; unknown histone marks	(138–140)
S100A4	TLR4, RAGE	Unknown; presumed mTOR-related	TRIM-like; similar cytokine upregulation to β-glucan	(65, 141)

**Figure 5:**
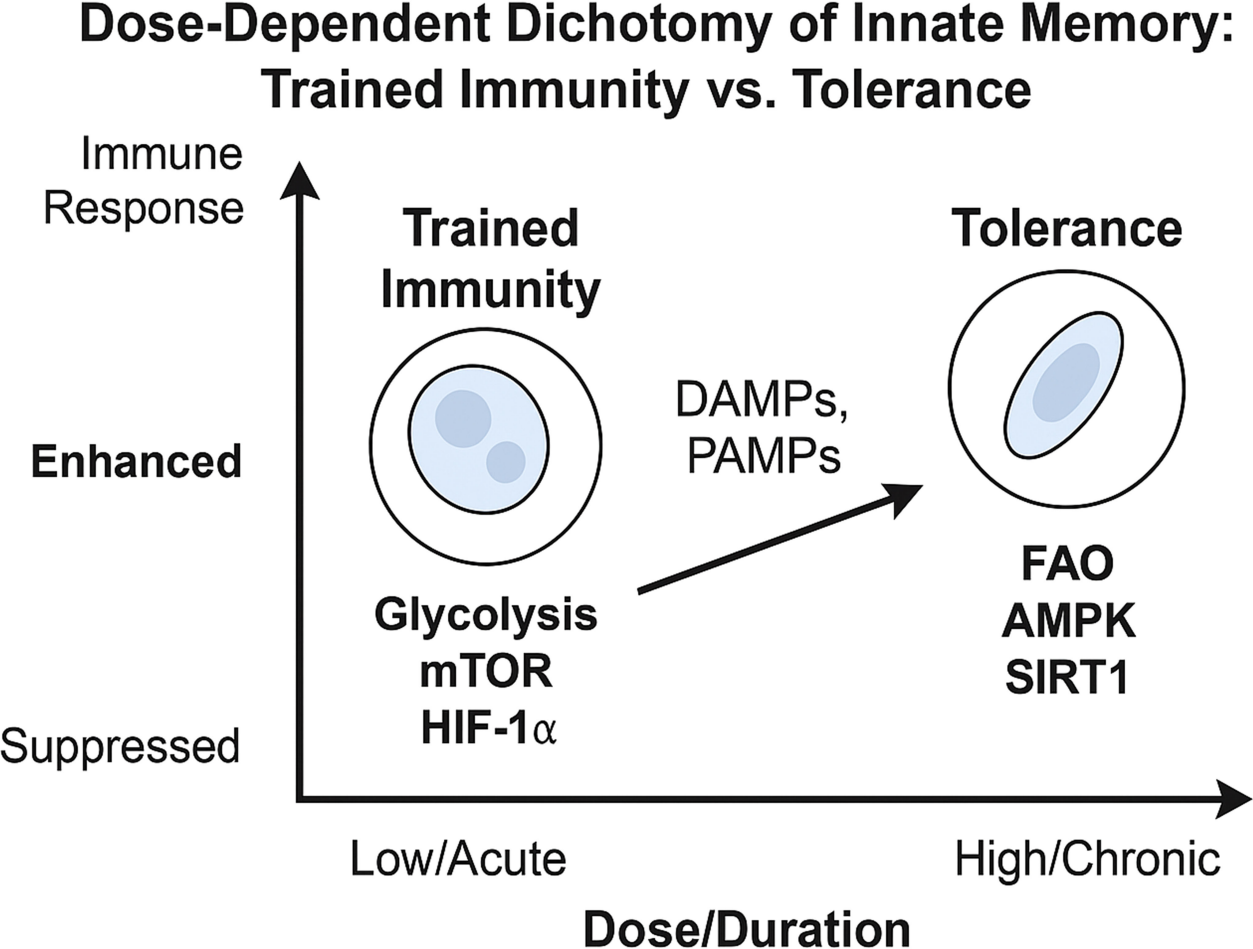
Dose-Dependent dichotomy of trained immunity vs. tolerance. This schematic depicts how low/acute vs. high/chronic doses of DAMPs (or PAMPs) drive innate cells toward either a trained or tolerant phenotype. Low-dose or acute exposure to PAMPs or DAMPs typically activates mTORC1 and HIF-1α–driven glycolysis, leading to trained immunity, characterized by enhanced cytokine production, increased metabolic activity, and epigenetic remodeling (e.g., H3K4me3, H3K27ac) that primes the cell for heightened responses upon secondary stimulation. High-dose or repeated stimulation instead promotes activation of AMPK, SIRT1, and autophagy pathways, resulting in immune tolerance. This state features suppressed inflammatory responses, increased fatty acid oxidation (FAO), and repressive epigenetic modifications (e.g., H3K9me3), which collectively dampen reactivity to subsequent challenges.

**Figure 6:**
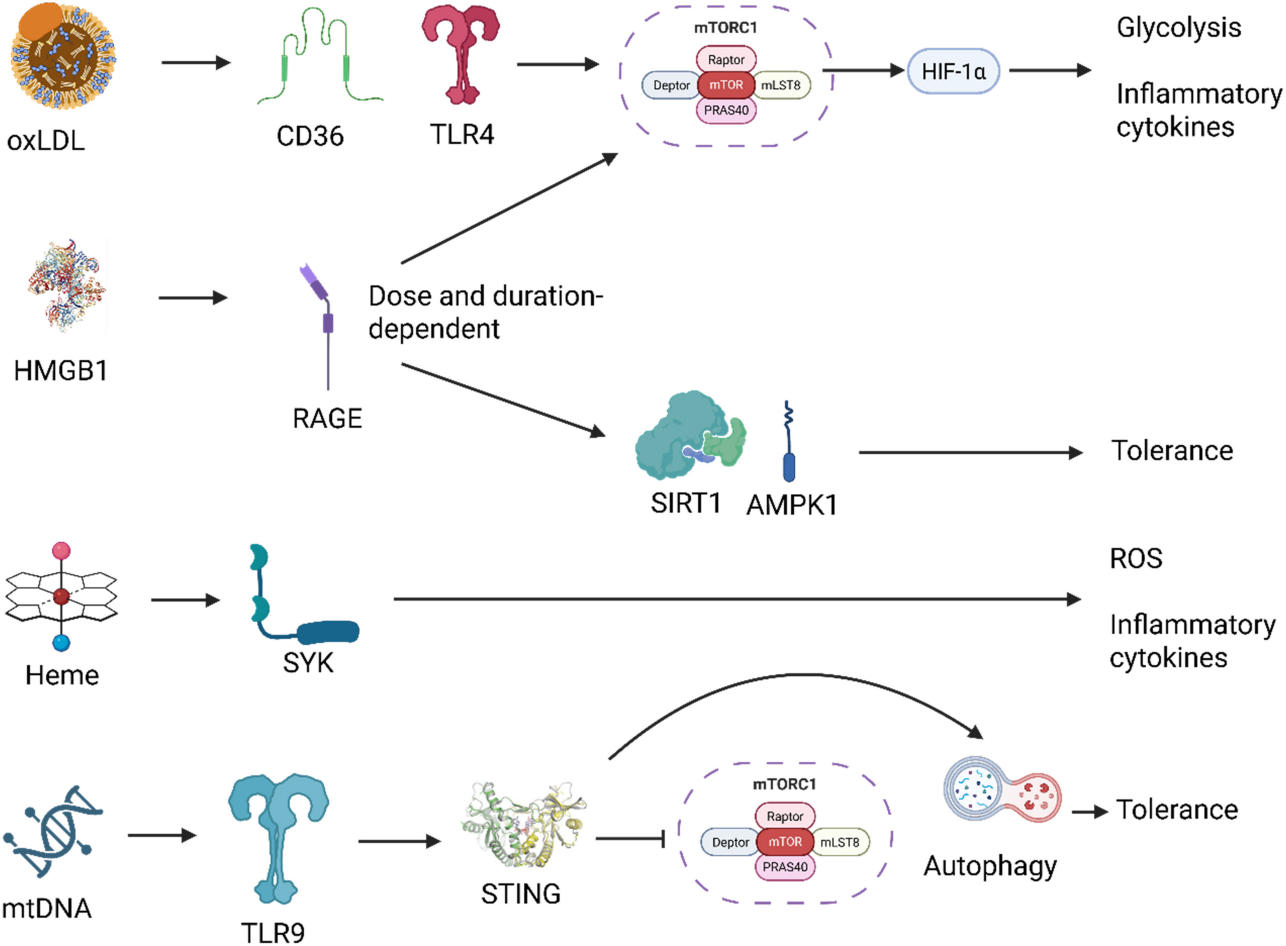
Distinct signaling pathways downstream of DAMPs mediate context-specific innate immune reprogramming. Damage-associated molecular patterns (DAMPs) activate unique receptor-mediated pathways that converge on distinct metabolic and transcriptional responses. For example, oxidized LDL (oxLDL) signals through CD36 and TLR4, activating the mTOR-HIF-1α axis and promoting trained immunity characterized by glycolysis and pro-inflammatory cytokine production. HMGB1, depending on dose and context, activates RAGE, leading to either AMPK/SIRT1-mediated tolerance or mTOR-driven training. Heme triggers SYK kinase signaling, driving reactive oxygen species (ROS) generation and a trained phenotype via a mTOR-independent route. Mitochondrial DNA (mtDNA) is sensed via TLR9, engaging the STING pathway, which intersects with autophagy and inhibits mTOR, promoting an autophagy-high, tolerant state. These divergent signaling routes illustrate how specific DAMPs imprint tailored innate immune memory programs depending on receptor usage, signal integration, and cellular context. Created with BioRender.com.

**Figure 7:**
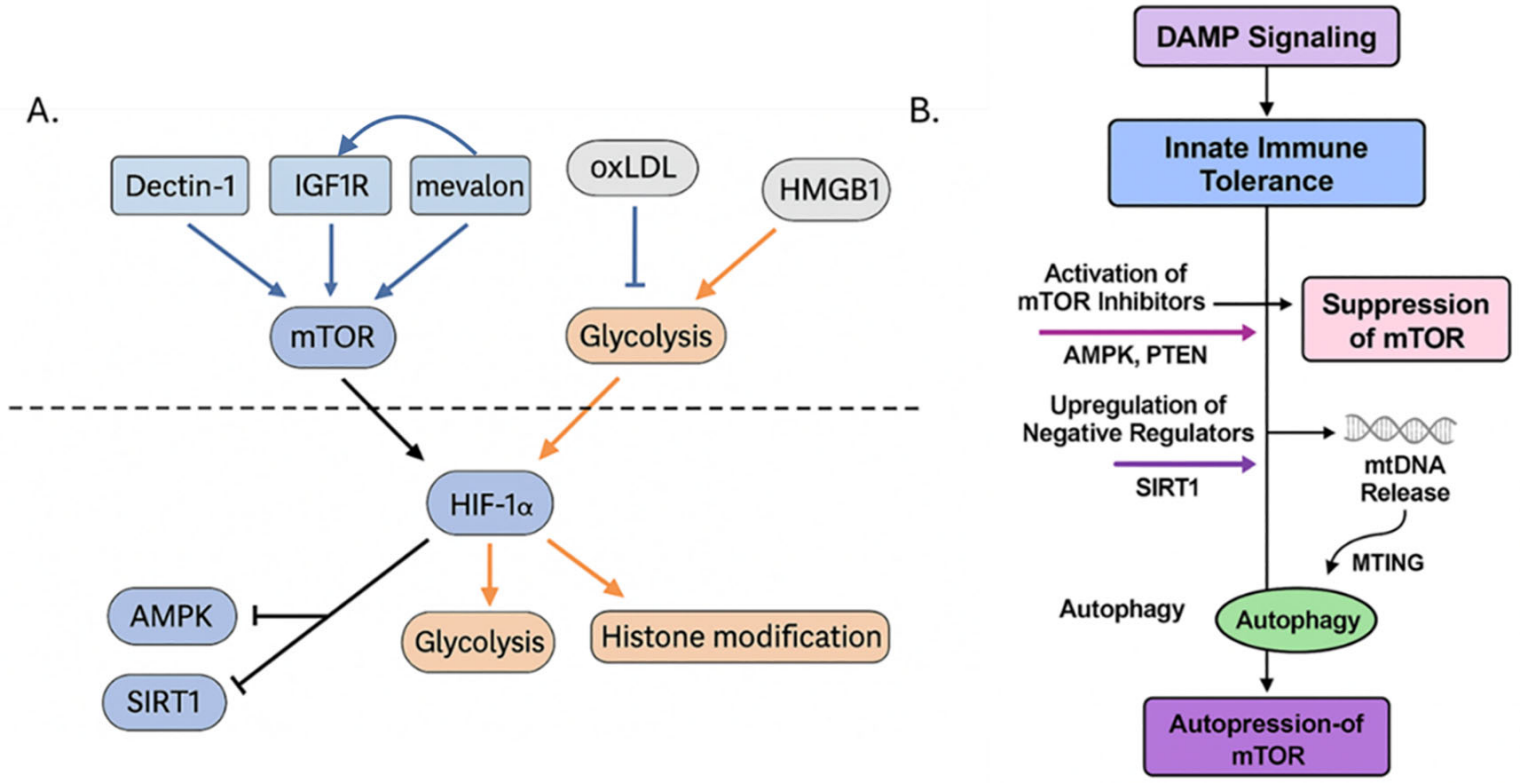
**(A)** The mTOR signaling axis integrates metabolic and epigenetic cues to regulate trained versus tolerized innate immune states. This diagram illustrates the central role of the mechanistic target of rapamycin (mTOR) in determining whether innate immune cells adopt a trained or tolerized phenotype following DAMP stimulation. Upstream activators such as oxLDL engages CD36/TLR4 and enhances IGF1R signaling via mevalonate (“mevalon”) accumulation, leading to PI3K–AKT–mTOR activation. Dectin-1, often engaged by fungal β-glucan and possibly some DAMPs, also activates mTOR via Syk and downstream AKT. Downstream of mTOR, activation of HIF-1α, glycolysis, and histone acetylation/methylation (e.g., H3K4me3, H3K27ac) leads to enhanced inflammatory gene transcription and the trained immunity phenotype. Various negative feedback and inhibitory loops exist; for example, in tolerized states, AMPK, SIRT1, and STING/autophagy pathways are activated by cellular stress, energy depletion, or chronic DAMP signaling (e.g., high-dose HMGB1 or mtDNA), which suppress mTOR activity. **(B)** Key inhibitory nodes are activated, such as AMPK and PTEN, which directly suppress mTOR activity, and SIRT1, are activated that are negative regulators that promote a tolerogenic program. In parallel, mitochondrial DNA (mtDNA) release during cellular stress activates STING and related pathways, which further inhibit mTOR through mitochondrial training-induced genes (MTING) and enhance autophagy. Together, these processes reinforce a feedback loop of autophagy-driven mTOR repression, leading to sustained immune tolerance and reduced inflammatory responsiveness. This promotes oxidative metabolism, histone deacetylation or repression, and reduced cytokine expression, resulting in immune tolerance. Created with BioRender.com.

An important consideration when discussing PAMPs/DAMPs driven innate memory is dosage (**Figure 5**). Low-level stressors such as LPS engage the dectin-1-mTOR-HIF-1α axis promoting resistance while high-level dosages can activate AMPK and induce tolerance (50). However, DAMPs by their characterization as disequilibrium endogenous molecules involve a dose-dependent consideration in their mechanism. Therefore, although DAMPs-dosing is important, the following section will focus primarily on the roles of different stressors in driving innate memory. Finally, although traditional understanding of DAMP-induced training excludes cytoplasmatic PRR activation (9), research into EVs provides an alternative mechanism for DAMP-induced TRIM involving pathways such as NLR and RIG-1. These EVs may provide an alternative training mechanism as PAMP-based models have shown that gut-derived EVs are linked to MyD88-mediated training of bone marrow-derived neutrophils (51). In the following sections we will explore the role of DAMPs in modulating epigenetic and metabolic reprogramming, and the pathways they may activate in establishing innate memory (**Figures 6**, **7**).

### 5.1 Oxidized LDL

Atherosclerosis involves the accumulation of oxidized lipids which have been shown to recruit and differentiate monocyte into macrophages within the intimal layer of the artery. It was first demonstrated that oxLDL could induce TRIM in monocytes with TLR4/TLR2 restimulation resulting in an upregulation of TNF-α and IL-6 as well as promoting CD36 and SR-A scavenger receptors resulting in the increase in foam-cell formation (8). Epigenetic analysis demonstrates H3K4me3 enrichment on promoters akin to β-glucan induced training that could be inhibited by methyltransferase inhibitors. Finally, the inhibition of PI3K and ERK could decrease TRIM. Other studies link innate memory of oxLDL with mTOR and NF-kB (52, 53). Considering that oxLDL-induced TRIM can be inhibited by using Torin1 and Raptor/Rictor siRNA gene silencing, this points to oxLDL-TRIM being activated by the Akt/mTOR/HIF-1α axis, a key pathway for β-glucan TRIM (54). However, subsequent studies highlighted an alternative mechanism where Liver X Receptor (LXR) activation can also induce TRIM phenotype (55). LXR agonists could augment oxLDL-induced TRIM while inhibitors reduced IL-6 and TNF-α cytokine response and H3K4me3/H3K27ac enrichment on promoters (56). These activators of HIF-1α result in a mevalonate/IGF1-R feedback loop known to drive TRIM epigenetic remodeling (45).

The “Warburg effect” glycolytic shift, increased cytokine production, and H3K4me3 and H3K27me3 enrichment on the TNF-α promoter is consistent in monocytes isolated from clinical patients with symptomatic atherosclerosis (57). Another focus of oxLDL has been in the Western Diet model with induction of NLRP3-mediated TRIM linked to the systemic inflammation characteristic of a Western Diet (21). Interestingly, a recent study demonstrates that a transient Western Diet followed by a healthier diet can induce TRIM which downregulates subsequent inflammatory response to DSS colitis and *Citrobacter rodentium*-induced colitis (58). OxLDL TRIM can impact intracellular steroid hormone regulation in monocytes with progesterone inhibiting oxLDL’s TNF-α and IL-6 TRIM response (59). Finally, studies note that epigenetic & metabolic rewiring of TRIM is not exclusive to innate immune cells with studies indicating that oxLDL can induce a mTOR/TLR2/TLR4-mediated TRIM phenotype in both aortic endothelial and coronary smooth muscle cells (60).

### 5.2 HMGB1 and vimentin

HMGB1 is a nonhistone protein from the nucleus that serves as a chaperone in the nucleus while promoting autophagy in the cytoplasm (61). HMGB1 can be released both actively as well as passively as a DAMP through various cell-death mechanisms such as pyroptosis, necrosis, and apoptosis. When interacting with PRRs TLR2 and TLR4, HMGB1 induces NF-kB dependent transcription (62). HMGB1 meanwhile binds with Mac-1 and RAGE to activate NF-kB and drive neutrophil recruitment in tissue injury (63). Incubation of splenocytes from healthy mice for 8 days with HMGB1, results in splenocytes that can elicit enhanced response to subsequent LPS stimulation (64). In a study comparing TRIM capability of multiple DAMPs to β-glucan, HMGB1 was found to exhibit TRIM properties albeit to a lower extent (65). While the mechanisms of HMGB1 and its different oxidation forms in inducing innate memory are uncertain, recent reports demonstrate how macrophages stimulated with dsHMGB1 present different transcriptomic profile to LPS and LPS/IFN-γ stimulated cells (66).

Vimentin meanwhile is an intermediate filament protein that acts on the dectin-1 receptor (67). Both endogenous vimentin and HMGB1 are released during transplantation and are a focus of post-organ transplantation ischemia-reperfusion injury TRIM. HMGB1 is upregulated during allogenic transplants in mice a week post-surgery (68). A 2018 paper from Braza et al. demonstrate that initial stimulation by vimentin followed by restimulation using HMGB1 could create a TRIM response that was similar to TRIM from β-glucan (69). In the same paper, the team developed rapamycin-based nanobiologics (mTORi-HDL), which could prevent innate training in an *in vitro* β-glucan monocyte model. Afterwards, they confirmed decreased restimulation by LPS in graft-infiltrating macrophages in mice from the nanobiologics following an allogeneic heart transplant. In studies, HMGB1 has been found to be enriched in EVs from both human and mice burn and express a biphasic immune response characteristic of burn immune dysfunction (26, 70). While no studies have yet linked the role that DAMPs-carrying EVs play in innate memory, more research is needed to understand the mechanisms in which these processes occur.

### 5.3 Hyperglycemia

In addition to causing complications such as atherosclerotic diseases and increased infection risk, evidence of the link between diabetes and inflammation has continued to grow over the years (71). In 1993, it was discovered that TNF plays a role in insulin resistance in mice, while epidemiological markers such as fibrinogen, IL-6, sialic acid, and C-reactive protein increase in humans with Type 2 diabetes (72). One driver of diabetes complications is hyperglycemia as transient levels of increased glycated hemoglobin levels correlate with long-term complications (73). This has resulted in the development of a concept known as “hyperglycemic memory”.

In a 2021 study by Thiem et al, STZ-treated hyperglycemic mice and *in-vitro* glucose stimulated/β-glucan co-stimulated monocytes demonstrate how glucose can exacerbate immune training and induce low levels of TRIM on its own with increased transcription of glycolysis-related genes (74). Furthemore, inhibition of the KMT2A epigenetic histone writers reduce TNF TRIM response (74). Hyperglycemia results in central innate memory training that can be adoptively transferred to healthy mice inducing a pro-inflammatory response mediated by the transcription factor Runx1 (75). Interestingly, glucose may also play a role in neurological innate training as *in vivo* murine Streptozotocin (STZ)-treatment can result in a TRIM-like inflammatory profile following LPS restimulation in brain microglia and astrocytes (76).

### 5.4 Heme

Heme is a tetrapyrrole that gets released by broken red blood cells following hemolysis or tissue damage. Heme can accumulate as “labile” heme in the plasma by containing a central iron atom that can change from a ferric (Fe3^+^) to ferrous (Fe2^+^) oxidation state. In severe cases when heme is unable to be managed, it results in free radical production, serves as a pro-oxidant, and can become a DAMP by activating the PRRs TLR4 and NLRP3. The classic TRIM monocyte-macrophage *in vitro* model was used to determine that specific PRR-recognized pattern of heme, not its oxidative properties, induces a characteristic TRIM immune response (9). The team notes H3K27ac epigenetic changes caused by heme through an alternative pathway to β-glucan. Unlike HMGB1 or oxLDL, TRIM induced by heme does not rely on mTOR with rapamycin treatment having no effect on training. Instead, SYK inhibition using R406 blocks heme-training corroborating other heme-related studies (77).

### 5.6 Monosodium urate crystals and uric acid

Urate serves as the final oxidation product in purine catabolism with high levels leading to the autoimmune gout pathologies. Moderate levels of urates are important for neuroprotective properties in Parkison’s, Alzheimer’s, multiple sclerosis, and amyotrophic lateral sclerosis (78). However, high levels of urate can result in nucleation of monosodium urate (MSU) crystals that can act as immunologically active DAMPs (14). The inflammation mechanism of urate involves priming by either MSU crystals or soluble urate to activate NF-kB through a MyD88-dependent pathway and initiate transcription of pro-IL-1β (79). Afterwards, MSU crystals induce the NLRP3 inflammasome pathway creating pro-caspase-1 which cleaves pro-IL-1β that in turn drives IL-1 mediated inflammation (80).

The effects of MSU and soluble urate results in downstream epigenetic/metabolic changes linked to innate priming which suggests the existence of TRIM-related training (81). Consistent with other mTOR training models, MSU crystals induce mTOR activation in monocytes as rapamycin or metformin treatment can inhibit inflammasome activity and subsequent inflammation (82). Epigenetically, Histone Deacetylase (HDAC) inhibition of MSU induced cells can downregulate MSU-induced proinflammatory cytokines (83). Meanwhile ChIP-seq analysis reveal that soluble urate can induce H3K27ac and H3K4me3 reprogramming in monocytes (81). Stimulation of PBMCs by uric acid followed by LPS restimulation results in improved IL-1β production *via* innate priming that is reversable through the use of methyltransferase inhibitors (84).

### 5.6 mtDNA/TLR9-mediated training

In shock-related disease models such as sepsis and trauma, mitochondrial DNA (mtDNA) has been linked to immunodysregulation (85, 86). In one model of mitochondrial herniation, release of mtDNA into the cytosol is mediated by BAK/BAX pores independent of apoptotic caspases (87). Similar to bacterial DNA, mtDNA contains unmethylated CpG’s that can target TLR9 and inflammasomes such as NLRP3 and AIM2 (86). Although no studies have yet linked mtDNA to innate memory, MyD88-mediated TLR9 has become a target of TRIM with findings that CpG-ODN can prime innate cells for improved clearance of *P. aeruginosa* infection (88). In a recent study it was found that TRIM from TLR9/MyD88-mediated CpG-ODN trained bone marrow derived monocytes can be adoptively transferred to wild type mice resulting in improved bacterial clearance (89). Like other TRIM models, mTOR was a key mediator in CpG-ODN TRIM with treatment by rapamycin inhibiting the trained phenotype. Although it is unclear whether CpG-rich mtDNA can result in similar training phenotypes, continued research can help elucidate the role of TLR9-mediated TRIM and mtDNA in shock-related disease models.

### 5.7 S100A4

S100A4 is a calcium binding protein that is involved with the process of tissue fibrosis. It acts via TLR4 and RAGE to upregulate proinflammatory cytokines such as IL-1 and IL-6 via a MyD88-mediated response (90). In a TRIM screening involving multiple DAMPs such as HMGB1, fibrinogen, and HSP90, only S100A4 was able to induce TRIM-like response at the levels of β-glucan (65). Further research needs to be done to understand whether DAMPs like S100A4 exhibit classical TRIM or if their enhanced immune response is a product of an elongated priming state.

## 6 Mechanisms of DAMP-specific metabolic reprogramming

DAMP exposure fundamentally alters the metabolic programming of innate immune cells, including how mitochondria handle the TCA cycle and OXPHOS. For example, oxLDL, a prototypical sterile DAMP in atherosclerosis, drives a strong mitochondrial response in trained cells. Monocytes briefly exposed to oxLDL show broad upregulation of mitochondrial genes and accumulation of TCA cycle metabolites during the training period with oxLDL-trained macrophages develop enlarged, hyperfunctional mitochondria, indicative of mitochondrial reprogramming (91). In contrast, other DAMPs may rely less on mitochondrial respiration. Aldosterone, an endogenous danger signal in cardiovascular stress, can induce trained immunity without significant changes in glycolytic flux or OXPHOS activity although persistent mTOR-dependent ROS production was found. This indicates that certain DAMP- stimuli achieve a trained state through alternative metabolic routes, such as fatty acid synthesis, discussed below. Many DAMPs that drive acute inflammation end up promoting glycolytic suppression if they persist. For example, prolonged exposure to HMGB1 or S100 alarmins can trigger autocrine IL-10 and metabolic brake mechanisms that shut down glycolysis over time (though acute HMGB1 is pro-glycolytic, chronic HMGB1 may lead to feedback inhibition) (92). In septic patients (who experience both PAMPs and DAMPs), monocytes often display a “glycolytic paralysis” consistent with endotoxin tolerance, which is linked to worse outcomes (93).

Many DAMPs also directly perturb cholesterol or fatty acid pathways to sustain the trained state. For example, oxLDL-conditioned monocytes adopt a long-lasting pro-atherogenic phenotype characterized by foam cell formation and amplified cytokine release (8). Mechanistically, oxLDL engages PRR that initiate these changes, such as minimally oxidized LDL binds to CD14 and TLR2/4 on monocytes, while heavily oxidized LDL binds to scavenger receptors like CD36 and LOX-1. These inputs drive cholesterol accumulation in macrophages (foam cell formation) and provoke inflammatory gene expression, creating a “primed” state. Trained immunity triggered by oxLDL is associated with increased cholesterol synthesis and storage as well as pro-inflammatory output. In fact, acute oxLDL exposure induces canonical trained-immunity traits: activation of mTOR-HIF1α signaling, elevated glycolysis, and histone H3K4 methylation at inflammatory gene promoters.

Cholesterol crystals also activate NLRP3, triggering IL-1β release that can act on hematopoietic stem cells to expand myelopoiesis (21). Indeed, as discussed above, Western diet failed to induce trained immunity in Nlrp3-deficient mice.

Aldosterone, a steroid hormone, was recently shown to *train* human monocytes *in vitro* by engaging the mineralocorticoid receptor and upregulating fatty acid synthesis enzymes. Transcriptomic and metabolic analyses revealed that fatty acid synthesis (FAS) is crucial for this trained phenotype: aldosterone-treated monocytes strongly induced expression of lipid anabolic enzymes. Inhibition of the fatty acid synthesis pathway (e.g. with an acetyl-CoA carboxylase inhibitor) completely abrogated aldosterone-induced trained immunity (94). At the chromatin level, aldosterone priming led to enrichment of the activating histone mark H3K4me3 at promoters of fatty acid metabolism genes highlighting how metabolic and epigenetic effects converge in this model. Notably, aldosterone-trained cells did *not* exhibit the typical glycolytic switch seen with many PAMPs; instead, glycolysis and OXPHOS remained unchanged while anabolic lipid pathways were elevated (95). This dependence on FAS is unique; while the PAMP β-glucan also causes some increase in fatty acid synthesis, inhibition of FAS does not block β-glucan-induced training, whereas it abolishes aldosterone-induced training. Aldosterone may also promote intracellular cholesterol loading in monocytes, compounding its pro-atherogenic effect.

A recent study by Wang et al. suggests that DAMPs can modulate lipid metabolism in innate cells, thereby influencing their inflammatory setpoint (96). In ulcerative colitis (a condition with chronic HMGB1 release), the DAMP HMGB1 was found to negatively regulate CPT1a (the rate-limiting enzyme for mitochondrial fatty acid oxidation, FAO) in macrophages. HMGB1 overexpression drove macrophages to reduce FAO and instead favor glycolysis and pro-inflammatory polarization. Knockdown of HMGB1 had the opposite effect, enhancing FAO and promoting an anti-inflammatory profile. These data suggest that high levels of certain DAMPs can actively suppress the tolerant metabolic program. In summary, tolerant innate cells exhibit increased fatty acid oxidation and mitochondrial support, which reinforces their low-inflammatory state.

Host-derived signals such as DAMPs also commonly utilize mTOR to rewire cellular metabolism toward a trained phenotype. Certain DAMPs, by engaging specific receptors, could feed into PI3K-Akt pathways that transiently activate mTOR (as seen with HMGB1 or IL-33 in acute inflammation), but the chronic presence of DAMPs often leads to feedback inhibition of mTOR. For instance, prolonged HMGB1-RAGE signaling can increase SIRT1 and AMPK activity in macrophages which tips the balance towards mTOR suppression. Mitochondrial DAMPs like mtDNA activate cytosolic stress responses (e.g. STING) that intersect with autophagy pathways, indirectly inhibiting mTOR and promoting a tolerant, autophagy-high state to dispose of damaged organelles (97, 98). The oxLDL-induced training of human monocytes as described above depends on persistent mTOR activation, as evidenced by mTOR phosphorylation and corresponding accumulation of HIF-1α and its target genes (54). Inhibition of mTORC1 by rapamycin or Raptor knockdown, oxLDL-trained monocytes no longer produce elevated TNFα/IL-6 upon a second stimulus. mTOR activity in this context was shown to drive ROS generation and a positive feedback loop, as mTOR-dependent cytosolic ROS further stabilized HIF-1α. In contrast, and discussed below, recent work showed that heme, another DAMP, induces trained immunity in macrophages independently of mTOR, relying instead on Syk kinase signaling (77) through an unknown mechanism. Endogenous metabolic signals themselves can feed into mTOR pathway. For example, that mevalonate/cholesterol pathway discussed above generates mevalonate which can act as a danger signal, activating IGF1 receptor and downstream PI3K-mTOR in monocytes (45). This implies an interesting autocrine loop, with a cell’s own metabolic output (mevalonate) reinforcing mTOR signaling and thus the trained state (**Figure 7**).

The role of mTOR in modulating immune cell functions during both the acute and chronic phases post-injury has been examined (99–101). Burn injuries induce a chronic hyper-responsive training state in the innate immune system, characterized by increased recruitment of neutrophils to the lungs and enhanced reactive oxygen and nitrogen species (RONS) production. Administration of rapamycin, reversed this hyper-responsiveness at time of injury, leading to decreased neutrophil oxidative burst and impaired bacterial clearance, thereby increasing mortality in burn-injured mice. This study underscores the critical role of mTOR in promoting long lasting neutrophil antimicrobial functions post-injury.

## 7 DAMPs and innate memory as possible drivers of immune dysfunction

As illustrated thus far, the mechanisms underlying innate memory are complex which is further complicated when translating *in vitro* research to clinical applications. In addition to vaccine-like promise in PAMPs-based TRIM through agents like β-glucan or BCG, in disease models such as sepsis there is an interplay between PAMPs and DAMPs that may modulate severity of disease progression. Evolutionarily adaptive DAMP-mediated inflammatory feedback loops that help to clear infections can become maladaptive when taken to extremes as suggested in Systemic Inflammatory Response Syndrome (SIRS) and Persistent Inflammation, Immunosuppression, and Catabolism Syndrome (PICS) models (102, 103). As demonstrated in oxLDL atherosclerosis and vimentin transplantation models, DAMPs are capable of inducing TRIM without a pathogen stimulus. The western diet has been a common target of DAMP-mediated TRIM.

“Dirty mice,” or mice exposed to diverse microbial environments, have emerged as valuable models for studying immune responses that closely resemble those in humans (reviewed in (104)). Unlike specific pathogen-free (SPF) mice, dirty mice possess an immune system that has matured through natural microbial exposures, leading to a more experienced and responsive immune profile. These recently developed platforms underscore the importance of considering environmental microbial exposures when studying trained immunity and its implications for disease models.

Translating DAMP-driven TRIM from mice to patients requires caution. Baseline myeloid populations differ with mice having lower circulating neutrophil fractions whereas humans are neutrophil-dominant with CD14/CD16 subsets (105). PRR thresholds are species-specific (e.g., TLR4/LPS sensitivity, Dectin-1 isoforms, inflammasome activation), so the same DAMP can bias toward training versus tolerance across species (106, 107). HSPC training shows species and age effects (G-CSF responsiveness, niche cytokines, lifespan of trained progeny (30, 108)), and human clinical modifiers such as sex, comorbidities, transfusions, sedatives, and drugs (statins, steroids, metformin) can either amplify or decrease TRIM signatures (21, 45, 109).

In addition to autoimmune frameworks, other DAMP-relevant TRIM have been proposed in sterile critical care models such as burns and trauma where sepsis-like paradigms are relevant. After burn injury, despite the return to more normal transcriptional activity following acute inflammatory states, macrophages and neutrophils exhibit a hyperinflammatory response when rechallenged by *Pseudomonas aeruginosa* (99). Other studies have demonstrated epigenetic reprogramming in circulating macrophages following trauma and burn while a recent study linked a central TRIM-mediated response to brain trauma that was shown to exacerbate inflammatory cardiac dysfunction (32, 110). Continued research into DAMP-driven TRIM in context of both autoimmune and critical care frameworks is crucial to be able to deliver future TRIM-targeting therapies. While mTOR activation enhances antimicrobial functions of neutrophils, contributing to improved pathogen clearance, it also predisposes to immune dysregulation and increased susceptibility to secondary infections during the chronic phase. These findings suggest that therapeutic modulation of mTOR signaling requires a delicate balance to harness its protective effects without exacerbating immune dysfunction. Further research is warranted to develop targeted interventions that can fine-tune mTOR activity, optimizing immune responses for better clinical outcomes in burn patients.

As research continues, integrating DAMP-related signals into the framework of innate immune memory will be crucial for devising interventions that can fine-tune immune responses in a range of sterile and infectious inflammatory conditions.

## 8 Discussion

### 8.1 Conceptual gaps-in-knowledge

As research continues, integrating DAMP-related signals into the framework of innate immune memory will be crucial for devising interventions that can fine-tune immune responses in a range of sterile and infectious inflammatory conditions. It is yet to be determined whether the outcome (training versus tolerance) is intrinsically dictated by the stimulus type; for example, exogenous signals, eg PAMPS (β-glucan, BCG vaccine) or DAMPs (oxLDL, heme) predominantly evoke a trained immunity phenotype, whereas others such as high-dose LPS invariably drive tolerance (39, 111, 112). An opposing view is that any PAMP or DAMP can lead to training or tolerance depending on exposure conditions; low/acute doses trigger a heightened responsive state, whereas high/repetitive doses induce a refractory state. This “dose-dependent” model is supported by evidence that even classical tolerogens like endotoxin can produce a trained response if given in subclinical amounts (50, 113). It remains unclear why certain DAMPs induce robust innate memory whereas others do not. Many sterile injury-associated DAMPs (e.g. mtDNA, ATP, nuclear histones) have not been shown to cause a lasting trained immunity response – some instead drive transient activation or tolerance. The field needs to determine what features of a stimulus (molecular structure, receptor engagement pattern, etc.) dictate the epigenetic outcome. One particularly understudied area is the role of mitochondrial danger signals in shaping innate immune memory. Mitochondria-derived DAMPs such as mtDNA, N-formyl peptides, and cardiolipin can mimic infection signals but recent data suggest they may push innate immunity toward a suppressed state; patients with acute myocardial infarction show elevated mitochondrial DNA in circulation associated with monocyte endotoxin tolerance (114). Do mtDAMPs actively reprogram monocytes via pattern recognition receptors (e.g. TLR9 for DNA) to become tolerant, and if so, through what epigenetic marks? Is this effect transient or long-lasting? It is also unknown whether mitochondrial signals could ever lead to a trained phenotype under different contexts; they have been mostly linked to immunoparalysis in studies to date. This represents a gap at the intersection of metabolism, cell death, and innate memory: deciphering how mitochondrial stress signals modulate myeloid training vs. tolerance is an important research priority. Another gap in our knowledge is how innate immune training interacts with the adaptive immune system. Innate training can influence the environment in which adaptive responses develop (for example, trained monocytes secrete more IL-1β, which could enhance T-cell activation). Conversely, tolerized innate cells produce more IL-10 and less IL-12, potentially skewing T-cell polarization towards regulatory or Th_2_ responses. Do trained macrophages or NK cells translate into better vaccine efficacy or improved heterologous immunity in humans (beyond correlative evidence)? Conversely, does a state of innate tolerance contribute to failures in vaccination or reactivation of latent infections? Bridging this gap will require integrated studies of innate and adaptive responses in models of infection, vaccination, and disease.

An important nuance is the dose- and context-dependence (“hormesis”) of innate memory and how this plays out in tissue-specific macrophage lineages. As outlined by Jentho and colleagues (115), low-level/short-lived danger signals can enhance cellular fitness and responsiveness, whereas high-level/chronic exposure skews toward adaptive suppression. Notably, functional readouts of ‘training’ are not uniform across lineages: while monocytes and neutrophils often display increased cytokine output and phagocytosis after training, microglia provide a counterexample in which phagocytic capacity is preferentially augmented in *tolerized* states. Work has demonstrated that repeated or chronic stimulation in the CNS can imprint an immune-depressed but debris-clearing microglial phenotype (116–118). These observations argue that outcome definitions (training vs tolerance) should be interpreted alongside stimulus dose and cell identity.

There is also debate about whether inducing innate immune memory is desirable or harmful. Trained immunity provides enhanced protection against infections and has been linked to improved outcomes (e.g. survival benefits of BCG or fungal exposure beyond specific pathogens). On the other hand, others point out that inappropriate or chronic activation of trained immunity by endogenous DAMPs may contribute to pathology, such as atherosclerosis (inflammatory foam cell formation from oxLDL-trained macrophages) and neurodegeneration (such as exacerbation of Alzheimer’s pathology by trained microglia (117)) Conversely, endotoxin tolerance can be protective by preventing hyperinflammation (as in sepsis), but if excessive it leaves the host immunosuppressed and susceptible to secondary infections. Another unresolved question is how the innate immune system balances repair mechanisms versus defense activation. Trained immunity skews cells toward inflammation (high IL-1β, TNFα, etc.), whereas tolerance reduces inflammation to facilitate recovery. For instance, after severe trauma or ischemia, DAMP-triggered tolerance might prevent further tissue damage by inflammatory overreaction – but at the cost of leaving the host open to infection (as seen in trauma and stroke patients with subsequent immune suppression) (119). Conversely, if DAMPs provoke trained immunity, they might clear debris and pathogens faster, but potentially at the expense of collateral damage and fibrosis. We do not know under which conditions DAMP-induced training is beneficial and when it becomes detrimental. Understanding this balance is critical for therapy, to know whether inducing a trained response in a sterile injury (e.g. heart attack) helps healing or worsens it through excess inflammation.

Current evidence points to reprogramming of bone marrow HSPCs to reconcile the fact that many innate cells have a short lifespan (days/weeks) yet innate memory can last for months or years. Such “central training” then continuously generate pre-trained myeloid cells (120). However, the mechanisms by which signals in peripheral tissues (or circulation) feed back to HSPCs are not fully elucidated. Cytokine mediators (like IL-1β, GM-CSF) and DAMP-induced flux of growth factors are implicated in conveying the memory imprint to the bone marrow niche, but many questions remain. How do tolerogenic marks (e.g. suppressed histone acetylation at inflammatory gene promoters) remain stable over time, and are they actively maintained by feedback loops or just slowly decay? A related issue is the potential fitness cost or trade-off for the stem cell pool; does carrying an epigenetic memory of past stress alter HSPC function or exhaustion over the long term? Key gaps include how long trained immunity lasts in people (some evidence suggests BCG effects persist for years, but how reliably)? and whether repeated or chronic exposures produce cumulative effects. For example, does an initial trained immunity from one insult (say, a mild infection or injury) alter the course of a completely different disease months later? Epidemiological hints (e.g. certain vaccines reducing all-cause mortality via innate mechanisms) are intriguing but mechanistically vague. Conversely, does a period of endotoxin tolerance (such as after severe sepsis) increase cancer risk or allow latent infections to flare? These are difficult questions to study, and thus gaps in knowledge. We also do not fully understand the reversibility/plasticity of innate memory states; can a tolerized immune system be “retrained” back to normal, and can a trained system be reset to avoid chronic inflammation? Addressing these gaps will likely require longitudinal human studies and novel experimental models that more faithfully replicate the human immune system (121).

### 8.2 Lack of biomarkers of training:

We currently lack readily measurable biomarkers of trained immunity or tolerance in clinical settings and developing a panel of innate memory biomarkers is an identified gap in our current knowledge (122). In the research lab, genome-wide histone modification patterns (like H3K27ac or H3K4me3 peaks at promoters) and transcriptomic profiles are used to identify trained *versus* tolerized cells. Metabolically, trained cells show increased glycolytic flux or certain metabolite accumulation (e.g. fumarate, succinate), whereas tolerant cells have high FAO and itaconate production. However, these signatures are difficult to assess in patients. There is a pressing need for verified surrogate markers that could indicate an individual’s innate immune state, such as specific cytokine patterns, surface markers, or metabolic indicators in blood tests. The lack of such biomarkers is a knowledge gap that hampers clinical translation. It also limits our ability to survey populations for trained immunity (e.g. after vaccination or diet changes) or to diagnose maladaptive tolerance (e.g. in sepsis). Research is ongoing to find candidate markers (such as circulating IL-1 receptor antagonist levels, or monocyte expression of certain cell-surface PRRs and checkpoint molecules in tolerance), but no consensus has been reached.

### 8.3 Therapeutic modulation of innate memory:

A future direction is the manipulation of trained immunity or tolerance as a therapeutic in various diseases. In cancer, for instance, activating trained immunity in tumor-associated macrophages or NK cells has potential to boost anti-tumor activity. There is interest in using compounds like beta-glucans or new synthetic agonists to reprogram innate cells in cancer patients towards a more inflammatory, tumoricidal state (123). On the other hand, chronic inflammatory and autoimmune diseases might benefit from inducing innate tolerance. For example, in conditions like gout or atherosclerosis where DAMP-driven trained immunity sustains pathology, drugs that promote a tolerogenic reprogramming of myeloid cells could alleviate disease (15). Early preclinical work shows that nanomedicines targeting monocytes can encourage tolerance and improve outcomes in organ transplantation models. In sepsis and critical illness, a two-pronged therapeutic strategy might emerge, with transient “training block” signals early on to avoid cytokine storm, then later promote immune training when patients become immunosuppressed. Immunomodulators like mTOR inhibitors (rapamycin) or AMPK activators (metformin) could be repurposed to tip the balance towards tolerance or training as needed as we have shown in burn injury (99). Given that metabolic reprogramming and epigenetic remodeling are at the heart of innate immune memory, future interventions may precisely target these processes. We anticipate further development of epigenetic drugs (inhibitors or enhancers of specific histone modifiers) to modulate trained immunity. For instance, blockers of the histone methyltransferases or demethylases that control key inflammatory gene loci might be used to prevent excessive training in inflammatory diseases (124). Conversely, HDAC inhibitors or bromodomain inhibitors could potentially be used in carefully titrated ways to sustain beneficial trained immunity in immunocompromised individuals. On the metabolic side, regulating the mTOR pathway is a clear avenue; transient mTOR inhibition might enforce a tolerant program (useful in cytokine storm or autoimmunity), whereas mTOR activation (for example via IGF1 or amino acid supplementation) might enhance training when needed for protection (though direct mTOR agonism is complex). Another target is the cellular metabolism of succinate, fumarate, and itaconate. These metabolites are known to influence immune memory. Drugs that mimic the effect of fumarate accumulation (which promotes training *via* HIF-1α stabilization) or that elevate endogenous antioxidative pathways (to modulate ROS and avoid excessive training) are under consideration (42). We expect to see trials of metabolic adjuvants or epigenetic therapies aimed at recalibrating innate immune memory in diseases ranging from sepsis to neurodegenerative disorders.

Another translational application of DAMP research will be strategies to manage the immune aftermath of sterile tissue injury (trauma, surgery, myocardial infarction, stroke). As discussed, increases of DAMPs in these settings can drive a profound innate immune tolerance, contributing to high infection rates in patients after injury (125). Future developments may include therapies to neutralize or clear DAMPs immediately after major tissue damage; for example, using extracellular DNA scavengers (DNAse enzymes to digest released mitochondrial DNA) or blocking DAMP receptors (such as antagonists to HMGB1 or IL-1 receptors), preventing an overshoot into tolerance. Monitoring patients innate immune status via biomarkers might enable personalized interventions; if a trauma patient shows signs of dangerous tolerance, one could administer trained immunity boosters (like recombinant GM-CSF or TLR agonists at low dose) to restore functionality. Conversely, if an individual is trending towards a hyper-trained, inflammatory state that could cause complications, targeted anti-inflammatory treatments could be applied. In essence, dynamic control of innate memory after sterile injuries could improve recovery and reduce complications. This precision medicine approach, treating innate immunity as a adjustable parameter, represents a forward-looking development as our understanding of DAMPs and innate memory deepens.

Lastly, future research is likely to adopt a more integrative view of innate immune memory across the lifespan and across systems. There is growing interest in how factors like aging, microbiome composition, stress, trauma and chronic metabolic status (e.g. obesity, diabetes) influence trained immunity versus tolerance. For example, aging is associated with “inflammaging,” possibly due to cumulative trained immunity and chronic low-grade DAMP release, and interventions like caloric restriction might mitigate this by promoting a more tolerant anti-inflammatory baseline (reviewed in (126)). We may see development of lifestyle or nutritional interventions that modulate innate memory (for instance, specific diets or microbiome-derived products that favor beneficial training without tipping into pathology). Additionally, cross-talk between different organ systems’ innate immunity (brain microglia, liver Kupffer cells, etc.) will be explored, which could open up new preventive strategies for complex diseases. The duality of trained immunity and tolerance provides a conceptual framework that future work will apply to diverse conditions from Alzheimer’s disease to cancer metastasis to vaccine responses in infants. By filling current knowledge gaps, the DAMP-driven innate memory can be translated into medical advances.
